# Combination of Radiofrequency Ablation With Resiquimod to Treat Hepatocellular Carcinoma *Via* Inflammation of Tumor Immune Microenvironment and Suppression of Angiogenesis

**DOI:** 10.3389/fonc.2022.891724

**Published:** 2022-06-02

**Authors:** Zhou Tian, Baojian Hong, Jianzhong Chen, Zhe Tang

**Affiliations:** ^1^ Department of Surgery, The Fourth Affiliated Hospital, International Institutes of Medicine, Zhejiang University School of Medicine, Yiwu, China; ^2^ Laboratory Medicine Center, Department of Clinical Laboratory, Zhejiang Provincial People’s Hospital (Affiliated People's Hospital, Hangzhou Medical College), Hangzhou, China; ^3^ Institute of Immunology School of Medicine, Zhejiang University, Hangzhou, China; ^4^ Key Laboratory of Immunity and Inflammatory Diseases of Zhejiang Province, Hangzhou, China; ^5^ Department of Surgery, The Second Affiliated Hospital, Zhejiang University School of Medicine, Hangzhou, China

**Keywords:** radiofrequency ablation, resiquimod, liver cancer, immune response, combination therapy

## Abstract

**Background:**

Radiofrequency ablation (RFA) destroys tumors through hyperthermic injury, which induces the release of immunogenic intracellular substrates and damages associated molecular patterns (DAMPs) to evoke a systemic immune response, but its therapeutic effect is limited. This study aimed to combine RFA with an immunomodulator, resiquimod (R848), to enhance the RFA-induced antitumor immunity.

**Methods:**

We performed RFA on subcutaneous tumors in immunocompetent mice and intraperitoneally injected R848 to observe the efficacy of the combination therapy. Our research investigated changes in the composition of tumor-infiltrating immune cells in primary and distant tumors by flow cytometry. Natural killer (NK) cell depletion experiment was applied to confirm the role of NK cell in the combination therapy. The expression levels of cytokines and chemokines were detected by real-time quantitative PCR. Immunohistochemical test was conducted to reveal tumor angiogenesis, tumor proliferation, and apoptosis after the different treatments.

**Results and Conclusion:**

Compared with RFA or R848 monotherapy, the combination therapy significantly slowed the tumor growth, prolonged the survival time, and shrank the tumor-draining lymph nodes of tumor-bearing mice. The flow cytometry results showed that tumor-infiltrating immune cells, total T cells, the ratio of CD8^+^ T and NK cells to CD45^+^ cells, and functional NK cells were obviously increased after the combined treatment. Distal tumor growth was also suppressed, and the profile of tumor-infiltrating immune cells was remodeled, too. In addition, the additive effect of the combination therapy disappeared after NK cell depletion. Furthermore, immunohistochemical results verified that R848 inhibited tumor angiogenesis in murine liver cancer, and the combination therapy promoted tumor cell apoptosis. In conclusion, our data suggest that RFA combined with R848 stimulated a stronger antitumor immune response and effectively inhibited liver cancer progression in a NK cell-dependent manner. Meanwhile, we confirmed that R848 inhibited tumor angiogenesis and promoted apoptosis in murine liver cancer. Overall, this is a promising therapeutic strategy to improve the efficacy of RFA in the treatment of liver cancer and provides a novel option for combined thermal ablation and immunotherapy.

## Introduction

Hepatocellular carcinoma (HCC) is one of the most common malignant tumors worldwide and the fourth leading cause of cancer-related deaths (1–3). East Asia and Africa are currently the regions with the highest incidence and mortality of HCC. The incidence and mortality of HCC in Europe and the United States have also been increasing in recent years (4, 5). At present, liver transplantation, surgical resection, and local ablation are the three major treatments for HCC (4, 6). However, the occult onset of HCC and the scarcity of donor livers severely limit the clinical application of surgical resection and liver transplantation (7–9). Local ablation therapy is especially suitable for these patients who are not suitable for surgery, and it is estimated that more than half of HCC patients have received local ablation therapy during their lifetime (10).

Radiofrequency ablation (RFA) is the most commonly used local ablation technique for HCC (11), and several studies have shown that RFA can achieve a similar therapeutic effect as surgical resection in the treatment of small HCC (single nodules ≤2 to 3 cm) (12–15). Interestingly, RFA induces tumor tissue coagulation necrosis and apoptosis, which lead to the release of immunogenic intracellular substrates to stimulate local anti-tumor immunity (16–20). Nevertheless, for a large tumor or a tumor located close to large blood vessels, RFA cannot completely destroy the tumor (incomplete ablation), and the residual tumor results in the recurrence and distant metastasis of HCC in the future (10, 21, 22). Therefore, it is clear that the anti-tumor immunity elicited by RFA monotherapy is too weak to effectively inhibit tumor recurrence and distant metastasis. In recent years, the combination of local ablation and immunotherapy for liver cancer is considered as a promising approach to boost RFA-induced immune response. Clinical trials of thermal ablation combined with immunotherapy, such as anti-PD1/anti-PDL1/anti-CTLA4 antibody, in the treatment of liver cancer have been widely carried out. Patients who received combination therapy had different degrees of improvement in overall survival or progression-free survival (23).

Resiquimod (R848) is a novel immunomodulatory agent which binds to Toll-like receptor7/8 and stimulates the release of various immunoregulatory cytokines, such as IFN-α, IL-6, and TNF-α, through MyD88-dependent or MyD88-independent pathways, thereby activating a cascade of signaling pathways to induce innate and adaptive immune response (24–26). Although R848 was originally used to study the role of antiviral and antibacterial immunity and the research on tumor has only started in recent years, several studies have shown that R848 significantly increases the number and function of CD8^+^ T cell and inflames the tumor immune microenvironment (TIME) (27–29). In addition, R848 has been used as an adjuvant in combination with anti-PD1/PDL1 antibody to treat colon cancer (30) and squamous cell carcinoma (31, 32) and achieved great therapeutic effects.

Here we demonstrated that the combined treatment of RFA and R848 not only ignited the TIME compared with RFA monotherapy but also increased the number and function of NK cell and CD8^+^ T cell and boosted the expression levels of multiple proinflammatory cytokines and NK cell-related chemokines in tumors. Meanwhile, we found that the combination therapy significantly inhibited HCC angiogenesis and proliferation but promoted tumor apoptosis (**Schematic Illustration**).

## Materials and Methods

### Cell Lines and Mice

Murine liver cancer cell line hepa1-6 was purchased from the cell bank of Chinese Academy of Sciences (Shanghai, China). The cells were cultured in Dulbecco’s modified Eagle’s medium (DMEM) containing 10% fetal bovine serum and 1% penicillin–streptomycin at 37°C and 5% CO_2_. The cells were digested for subsequent use when they reached 70% density.

Six- to 8-week-old male C57BL/6 mice were obtained from Shanghai SLAC Laboratory Animal Co. Ltd. Hepa1-6 cells (4 × 10^6^) were resuspended in 100 ul phosphate-buffered saline (PBS) and were subcutaneously injected into the right flank of C57BL/6 mice. In the abscopal effect assay, hepa1-6 tumor cells (3 × 10^6^) were simultaneously inoculated into the bilateral flanks of the mice. Tumor progression was measured with a vernier caliper. Tumor volume was calculated with the following formula: (length × width^2^)/2. At about 1 week later, the tumor-bearing mice were randomly divided into four groups to receive different treatments. All animal experiments followed relevant experimental animal ethic requirements and were approved by the Laboratory Animal Welfare Ethics Review Committee of Zhejiang University.

### Radiofrequency Ablation Therapy

For the radiofrequency ablation (RFA) and RFA+R848 groups, the mice were anesthetized with ketamine + xylazine solution (i.p., 90 + 8 mg/kg). After the mice were fully anesthetized, the abdominal hair was shaved, and they were fixed on the electrode plate in prone position; then, 1 ml PBS was sprayed on the contact area between the electrode plate and the mouse’s skin to increase the conductivity. A 480-kHz RFA generator (S-1500, MedSphere, Shanghai) was then connected, and a 17-gauge monopolar electrode was inserted along the long axis of the tumor so that the electrode tip reached the center of the tumor, and the ablation was performed with parameters of 5 W and 15 s. For the control and R848 groups, the mice received the same treatments but with the RFA generator turned off. After the RFA procedure, all mice were resuscitated on a 37°C blanket. A picture of the tumor RFA model is shown in **Supplementary Figure S6**.

### Resiquimod Therapy

The powder of resiqumod (R848) was obtained from MedChemExpress (Monmouth Junction, NJ, USA). The powder was prepared into a solution according to the manufacturer’s instructions and filtered with a 0.2-um syringe filter before administration. For the mice in the R848 and RFA+R848 groups, R848 solution (1 mg/kg,100 ul) was intraperitoneally injected on the same day as with the RFA treatment and then once every 2 days until the mice were sacrificed or dead. For the control and RFA groups, the mice were injected in the same way with a control solution without R848.

### Flow Cytometry

Flow cytometry was performed to analyze the tumor-infiltrating immune cells. In brief, tumors were peeled off the skin after the mice were sacrificed. The edge of the tumor was cut into 1-mm^3^ size, and then the fragments were placed for an hour in 5 ml DMEM containing 0.1 mg/ml DNase-I and 1 mg/ml collagenase IV at 37°C to get a single-cell suspension. Next, the cell suspensions were filtered through a 70-μm strainer to filter out incompletely digested residues. For cell membrane staining, Zombie Aqua Fixable Viability Kit was first applied, according to the manufacturer’s instructions, to distinguish live cells from dead cells, and then a suspension of various antibodies was used for cell membrane staining. For intracellular cytokine staining, the cells need to be stimulated with Cell Activation Cocktail (with Brefeldin A) first, followed by live–dead staining, cell membrane staining, fixation and permeabilization, and incubation of anti-IFN-γ and anti-Granzyme B monoclonal antibodies with cell suspensions. After dyeing, the excess dye was washed off with PBS, and the suspension was filtered again with a 40-μm nylon mesh to get the final single-cell suspension. All samples were acquired on BD LSR Fortessa (BD Biosciences), and data was analyzed with FlowJo 10.5.3 software (FlowJo LLC, Ashland, USA). The gating strategy is provided in **Supplementary Figure S2**.

The antibodies and reagents used in the flow cytometry analysis were obtained from Bio-Legend (San Diego, CA, USA), namely: Zombie Aqua Fixable Viability Kit, BV605-conjugated anti-CD45, APC-conjugated anti-CD3, Perp-Cy5.5-conjugated anti-NK1.1, PE-conjugated anti-CD4, FITC-conjugated anti-CD8, APC-conjugated anti-TCRβ, PE-conjugated anti-CD11b, BV711-conjugated anti-Ly6C, BV650-conjugated anti-Ly6G, APC-conjugated anti-F4/80, APC-Cy7-conjugated anti-I-A/I-E (MHCII), APC-Cy7-conjugated anti-CD45R (B220), PE-Cy7-conjugated anti-CD11c, FITC-conjugated anti-CD86, PE-Cy7-conjugated anti-IFN-γ and FITC-conjugated Granzyme B, Cell Activation Cocktail (with Brefeldin A), fixation buffer, and permeabilization wash buffer.

### NK Cell Deletion

The tumor-bearing mice were divided into three groups, and then they received RFA, RFA+R848, or RFA+R848+anti-NK1.1 antibody (Bio-Legend, San Diego, CA, USA). R848 was administered as previously described. Anti-NK1.1 (300 μg/mouse) or sham antibodies were injected intraperitoneally into the mice starting on the day before the RFA and then once every 3 days—for a total of 3 injections. Detection of NK cell in mouse blood was performed using flow cytometry to verify the efficiency of NK1.1 antibody.

### Real-Time Quantitative PCR

Approximately 0.1 g of tumor tissue was homogenized, and then total RNA was extracted using RNeasy Mini Kit (Qiagen, Germany), according to the manufacturer’s instructions. The synthesis of cDNA from RNA was achieved with Prime-Script™ RT reagent kit (TaKaRa). qPCR was performed on LightCycler 480 II system (480II-384, Roche, Germany) in a 10-µl reaction mixture containing SYBR Green I (Yeasen, Shanghai). The parameters are set to 40 cycles of 95°C for 15 s, 60°C for 15 s, and 72°C for 30 s. The expressions of the desired genes were normalized to GAPDH, and data were further analyzed by the 2^−ΔΔCT^ formula. The final results are presented as fold change to the control group, and the primer sequences are provided in **Supplementary Table S1**.

### Immunochemistry Staining

Tumor tissues were fixed in 10% neutral buffered formalin and then embedded in paraffin. For immunochemistry (IHC), the paraffins are cut into 4-μm slices. In the deparaffinization of sections to water, xylene and various concentrations of ethanol (100, 95, 85, and 75%) were used. Endogenous peroxidase was inactivated, antigen was retrieved, followed by goat serum blocking and incubation with primary anti-CD31 (AF3628, Bio-Techne) antibody, anti-VEGFA (19003-1-AP, Proteintech) antibody, anti-cleaved-caspase3 (9661L, Cell Signaling) antibody, and anti-ki67 (12202S, Cell Signaling) antibody. Next, the samples were incubated with a horseradish peroxidase-conjugated secondary antibody, and a color developer (diaminobenzidine) was used to develop color at an appropriate concentration. Finally, five fields of each section were randomly selected at ×400 magnification for counting of positive cells, and for ki67 staining, the integrated optical density value was calculated by ImageJ software (https://imagej.nih.gov/nih-image/) under the same threshold conditions.

### Statistical Analysis

All data analyses were performed by GraphPad Prism 8.0.2 (GraphPad Software, Inc.), and *p*-value <0.05 was regarded as statistically significant. The detailed statistical methods are presented in the figure legend.

## Results

### Combination of RFA With R848 Constrains the Growth of HCC and Extends the Survival of Tumor-Bearing Mice

**Figure 1:**
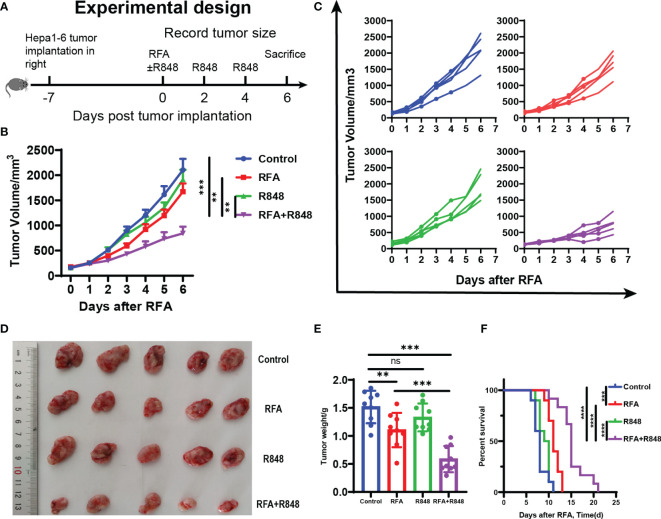
Potent antitumor efficiency following radiofrequency ablation combined with resiquimod. **(A)** Diagram of the experimental design for assessment combo treatments in mice liver cancer model (hepa1-6). **(B)** Tumor growth curves of mice that received different treatments. **(C)** Tumor growth in each mouse after different treatments. **(D)** Representative image of hepa1-6 tumor excised at day 6. **(E)** Quantitative analysis of tumor weight at sacrifice in different groups; summary analysis of the result of the two experiments. The experiments represented in **(B–E)** were repeated 3 times, with 4–6 mice per group. All data are shown as mean ± SEM, and two-tailed Student’s *t*-test was performed to compare the statistical differences between the two groups. **(F)** Kaplan–Meier overall survival analysis (*n* = 10 for each group), and this experiment was repeated 2 times. Statistical significance was evaluated by log-rank (Mantel–Cox) tests. **p < 0.01; ***p < 0.001; ****p < 0.0001; ns, not significant.

To evaluate whether RFA, R848, and the combo treatment elicit an effective anti-tumor immunity, hepa1-6 liver cancer cells were subcutaneously implanted into the right flank of C57BL/6 mice. At 1 week later, the mice were randomly assigned to four groups when the diameter of the tumor has reached 6–8 mm. The mice in the four groups received no treatment (control), RFA treatment, R848 treatment, and combined treatment (RFA+R848), respectively. Tumor volume was recorded every day, and tumor growth curves were plotted (**Figure 1A**).

As shown in **Figures 1B–D**, in contrast to the control or monotherapy group, the tumors of mice treated with RFA+R848 grew significantly slower and were smaller at the end of the experiment, whereas only a negligible tumor growth inhibitory effect was detected in the RFA and R848 groups. Combo treatments especially distinctly constrained the growth of tumor in the first 3 days after RFA. The tumor growth kinetics of mice that received different treatments is shown in **Figure 1C**. Both the RFA and combo treatments evidently reduced the tumor weight compared with the control group, but the tumor in the RFA+R848 group was significantly lighter than that in the RFA group (**Figure 1E**). Besides this, we observed that the tumor-draining lymph node in the RFA+R848 group was a little bit smaller than that in the control group, but there was no significant difference between the RFA and control groups (**Supplementary Figure S1A**). In addition, spleen size was not different among the groups (**Supplementary Figure S1B**). On the other hand, we also showed that combo treatments obviously extended the survival of tumor-bearing mice compared with those from the monotherapy or control group (**Figure 1F**). The median survival for mice treated with RFA+R848 was 15 days, an increase of 36.4 and 57.9% compared with the RFA (11 days) and R848 (9.5 days) group, respectively. We additionally found that RFA alone can also slightly improve the survival time of tumor-bearing mice.

### Combination of RFA With R848 Modulates the Profiles of Tumor-Infiltrating Immune Cells

**Figure 2:**
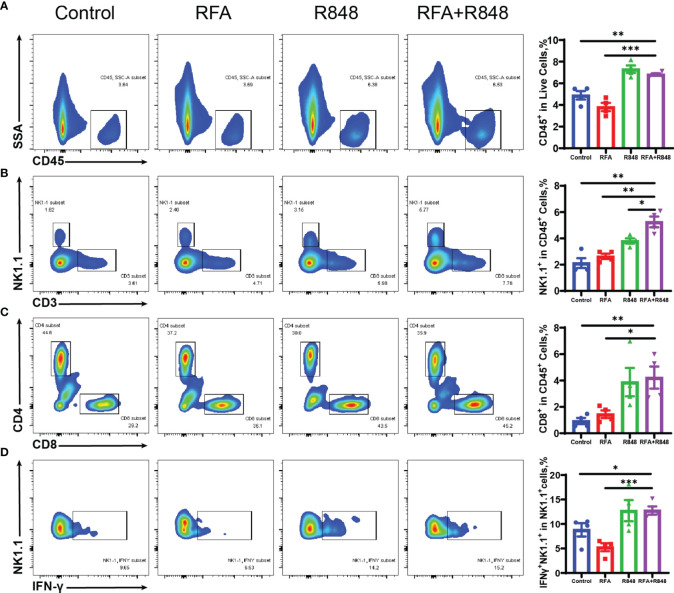
The composition of tumor-infiltrating immune cells is changed after combination therapy. **(A)** Ratio of tumor-infiltrating immune (CD45^+^) cells to live cells in different groups; quantitative analysis results are shown on the right. **(B)** Representative flow cytometry plots of the percent of tumor-infiltrating NK (CD3^-^NK1.1^+^) cells in CD45^+^ cells and the corresponding quantitative results. **(C)** Intra-tumoral CD8^+^ T cells were gated from CD3^+^ cells, and then the ratio of CD8^+^ to CD45^+^ was calculated; %CD8^+^ T in CD45^+^ cells = %(CD8^+^CD3^+^CD45^+^/CD45^+^). **(D)** The proportion of IFNγ-secreting NK (IFNγ^+^NK1.1^+^) cells to total NK cells in the tumors of mice that received different treatments. The experimental results from one of the two independent experiments are shown; *n* = 4 per group. Statistical comparison was performed by two-tailed Student’s t-test. All error bars represent mean ± SEM. *p < 0.05; **p < 0.01; ***p < 0.001. IFNγ, interferon γ; NK, natural killer.

We then revealed the effect of the combo treatments on tumor-infiltrating immune cells by flow cytometry (the gating strategy is shown in **Supplementary Figure S2**). Mice that received different treatments were executed on the 6th day, the tumors were peeled off the skin, and the edge of the tumor was cut into pieces to get single-cell suspensions. As shown in **Figure 2A**, there was a distinct increase in intra-tumoral CD45^+^ immune cells after the combined treatments compared with the control or RFA monotherapy, but no significant difference was found between the RFA+R848 group and the R848 group.

Natural killer (NK) cells and CD8^+^ T cells play an important role in liver cancer progression (33–36). We found that the combination therapy clearly elevated the ratio of NK, CD3^+^T, and CD8^+^ T cells to CD45^+^ immune cells compared with those in the control or RFA monotherapy (**Figures 2B** **, C**; **Supplementary Figure S3A**). Furthermore, the proportion of NK cells in the combined treatment group is significantly higher than in the R848 group, whereas the ratio of CD8^+^ T cells is not. In addition, the proportion of IFNγ^+^ cells in NK cells in the combined treatment group is higher than that in the RFA or control group (**Figure 2D**). Only a slight increase was observed after the combo treatments in terms of the frequency of IFNγ^+^ CD8^+^ T cells compared with no treatments, and there was no statistical difference between the combined group and the RFA group (**Supplementary Figure S3D**). Besides this, the ratio of Granzyme B^+^ cells in NK cells and CD8^+^ T cells was not different among the four groups (**Supplementary Figures 3E** **, F**). This implies that the combinational effect is mainly attributed to NK cells.

**Figure 3:**
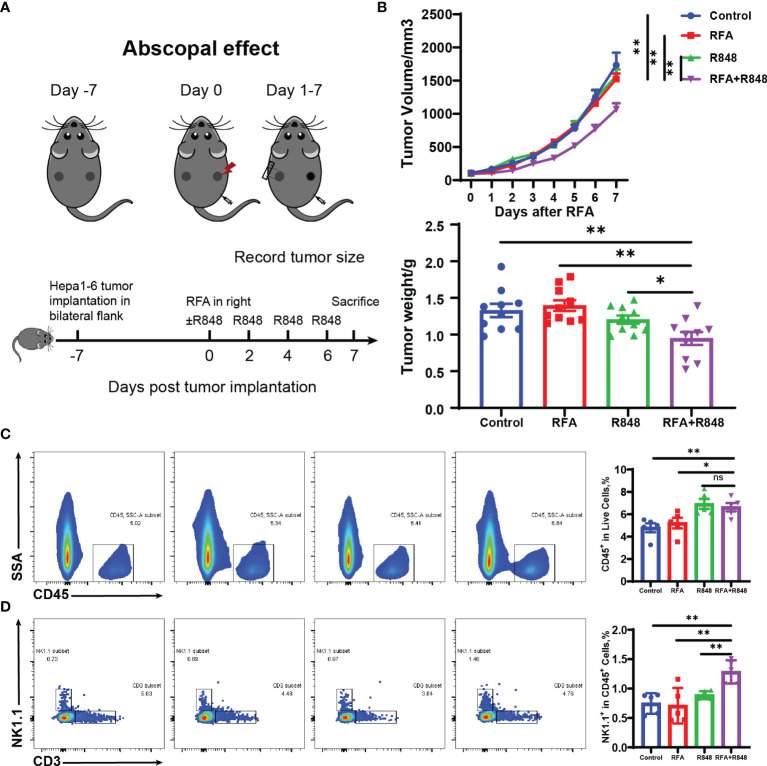
Systemic antitumor immune response is elicited after the combination of radiofrequency ablation with resiquimod. **(A)** Schematic diagram of the experiment to understand the different operations at different points in time. **(B)** Distant tumor growth curves of tumor-bearing mice treated with different approaches and the tumor weight (from two independent experiments) on the untreated side when the mice were sacrificed. **(C, D)** Tumors from the untreated side were dissected and made into single-cell suspensions for flow cytometry. The representative flow cytometry plots of tumor-infiltrating immune (CD45^+^) cells and natural killer (NK; CD3-NK1.1+) cells as well as the relative quantification of the ratio of CD45^+^ cells to live cells and the proportion of NK cells to CD45^+^ cells are shown. The results represent one of two independent experiments; *n* = 5 in each group. Statistical analysis was performed by two-tailed Student’s *t*-test. All error bars represent mean ± SEM. *p < 0.05; **p < 0.01; ns, not significant.

The flow cytometric assay also detected that the proportion of CD4^+^ T cells, B cells (CD3^-^CD45R^+^), macrophages (Ly6c^-^CD11B^+^F4/80^+^), monocytes (CD11B^+^MHCII^-^Ly6c^+^Ly6g^-^), and neutrophils (CD11B^+^MHCII^-^Ly6c^+^Ly6g^+^) to CD45^+^ immune cells was not different among the four groups. Moreover, there was also no difference between the ratio of M1 (MHCII^+^ macrophage)/M and M2 (MHCII^-^ macrophage)/M (**Supplementary Figures S3B, C**, **S4A–E**). However, interestingly, similar to the role of R848 in pancreatic cancer (27), the percentage of dendritic cells (DCs, CD11B^+^MHCII^+^CD11C^+^F4/80^-^) as a percent of CD45^+^ cells in both the R848 group and the RFA+R848 group was significantly reduced, while there was no difference in the proportion of CD86^+^ DCs to the total DCs between groups (**Supplementary Figures S4F, G**).

### Abscopal Effect Is Induced by RFA+R848 Treatments

To investigate the systemic antitumor immune response of combo treatments, we simultaneously implanted hepa1-6 liver cancer cells into the bilateral flanks of C57BL/6 mice. After a week, the mice were divided into four groups when the tumors have reached approximately 100 mm³. The tumor on the right flank was regarded as the primary tumor for RFA therapy, while the contralateral tumor was considered the distant tumor for monitoring and flow cytometry analysis. R848 was injected intraperitoneally once every 2 days—for a total of 4 injections. The mice were sacrificed on the 7th day after RFA (**Figure 3A**). The volume and weight of distant tumors manifested that only a negligible tumor growth inhibition was induced by RFA or R848 monotherapy. Nevertheless, the RFA+R848 treatment significantly reduced the tumor burden of the tumors that were left untreated (**Figure 3B**). Corresponding to these results, the flow cytometry analysis revealed that the combo treatments showed a stronger ability to increase the frequencies of both CD45^+^ and NK cells in the distant tumors compared with the control or RFA treatment. Furthermore, the proportion of NK cells to CD45^+^ cells in the combination therapy group was highest among the four groups (**Figures 3C** **, D**). This phenomenon further proves the role of NK cells in combination therapy. However, unlike a tumor *in situ*, no difference was detected in the ratio of CD3^+^ and CD8^+^ T cells to CD45^+^ cells in distant tumors among the four groups. The proportion of CD4^+^ T cells was not changed either (**Supplementary Figure S5**). What is more, similar to the primary tumor, except for DCs, the tumor-infiltrating myeloid immune cells in mice with different treatments almost did not differ in the distant tumor. The data is not presented.

### NK Cells Are Essential for the Antitumor Immunity Elicited by the Combined Treatments

**Figure 4:**
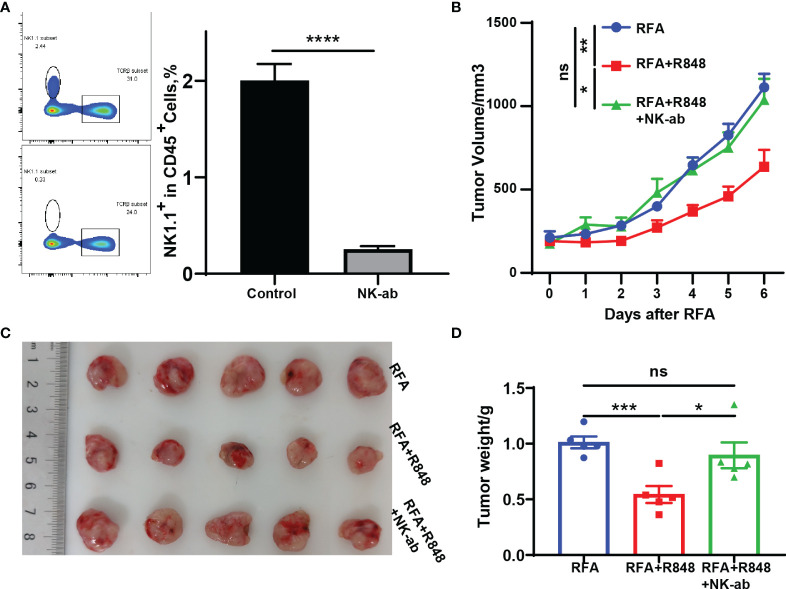
The superimposed effects induced by combination therapy are natural killer (NK) cell dependent. **(A)** NK1.1 antibodies were injected intraperitoneally at 1 day prior to radiofrequency ablation (RFA) and once every 3 days until the mice were sacrificed at 3 days after injection tail vein blood was collected to verify the clearance efficiency. **(B)** Tumor growth curves of mice depleted for NK cells and treated with RFA + resiquimod. **(C, D)** Tumor weight and a representative picture of the tumor excised at sacrifice. One of the two independent experiments is shown; *n* = 5 in each group. Statistical analysis was performed by two-tailed Student’s *t*-test. All error bars represent mean ± SEM. *p < 0.05; **p < 0.01; ***p < 0.001; ns, not significant.

On the basis of the research that we have made, those mice that received the combination therapy not only had the highest proportion of NK cells among the four groups but also had enhanced NK cell function (**Figures 2B** **, D**). Hence, to directly confirm the role of NK cells in combination therapy, we performed a NK depletion test. Anti-NK1.1 antibody was injected intraperitoneally at 1 day before the RFA and then once every 3 days until the mice were sacrificed. The rest of the operation was similar to **Figure 1A**. The depletion efficiency is shown in **Figure 4A**. The tumor growth curves indicate that the RFA+R848 treatment evidently reduced the tumor burden compared with RFA monotherapy, whereas the inhibition of tumor growth disappeared in the absence of NK cells (**Figure 4B**). Correspondingly, the tumors in the RFA+R848 group were the lightest and smallest among the three groups, but the tumor size was not reduced after using anti-NK1.1 antibody (**Figures 4C** **, D**).

### RFA+R848 Treatment Promotes the Expression of Lymphocyte-Related Cytokines and NK Cell-Related Chemokines in Tumor Tissue

**Figure 5:**
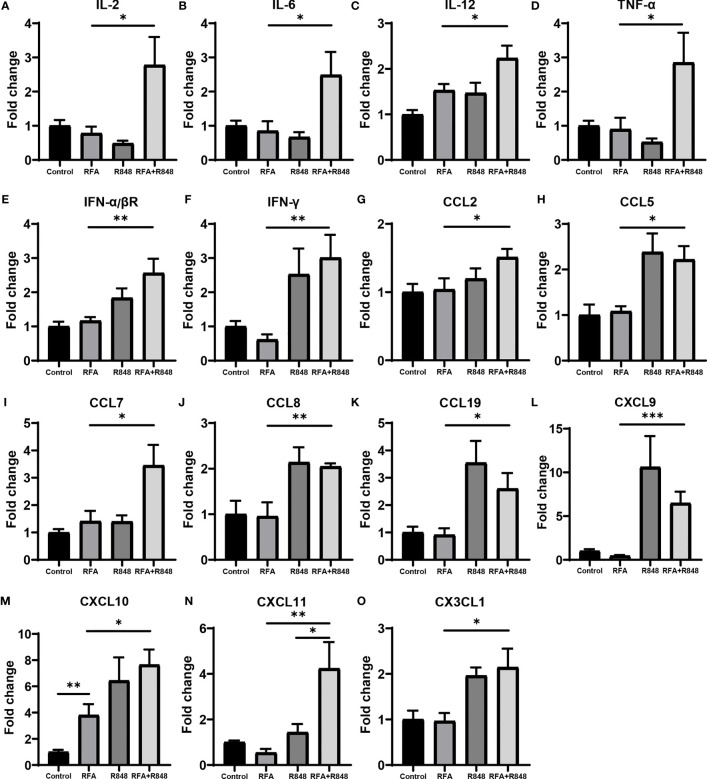
The profile of cytokines and chemokines are remodeled after radiofrequency ablation and resiquimod treatments. **(A**–**C)** qPCR detected the intra-tumoral mRNA expression of cytokines associated with the activation of T cells and natural killer (NK) cells following different treatments. **(D**–**F)** Expression levels of IFN-α/βR and cytokines related to the function of T cells and NK cells. **(G**–**O)** Expression of multiple chemokines with chemotaxis to NK cells. All results are presented as fold change relative to the control group. One of the two independent experiments is shown; *n* = 7–10 per group. Statistical analysis was performed by two-tailed Student’s t-test. Error bars represent mean ± SEM. *p < 0.05; **p < 0.01; ***p < 0.001.

Various cytokines and chemokines play a crucial role in the tumor microenvironment (24, 37–39), and chemokine networks are essential for NK cells exerting antitumor effects in solid tumor (40). We discovered that the expression of IL-2, IL-6, and IL-12 in these tumors, associated with the activation of T cells and NK cells, was evidently increased after the combo treatments (**Figures 5A** **–C**). At the same time, the expression of IFN-α/βR and cytokines related to the function of T cells and NK cells, such as TNF-α and IFN-γ, was also significantly increased after the combination therapy (**Figures 5D** **–F**). This inflammatory phenomenon is a reflection of the widespread and powerful immune activation within the tumor microenvironment and is consistent with the changes that we observed earlier in tumor-infiltrating lymphocytes. Although NK cells express many chemokine receptors, CCR2, CCR5, CCR7, CXCR3, CX3CR1, and their ligands are thought to play a major role in attracting NK cells to infiltrate tumors (40). As depicted in **Figures 5G–O**, the expression levels of the ligands corresponding to the aforementioned chemokine receptors in the RFA+R848 group were markedly higher than those in the RFA group. In summary, these multivariate data suggest that the combination therapy evidently reshaped the HCC immune microenvironment, leading to the inhibition of tumor growth.

### Combo Treatments Suppress Angiogenesis and Promote the Apoptosis of Liver Cancer

**Figure 6:**
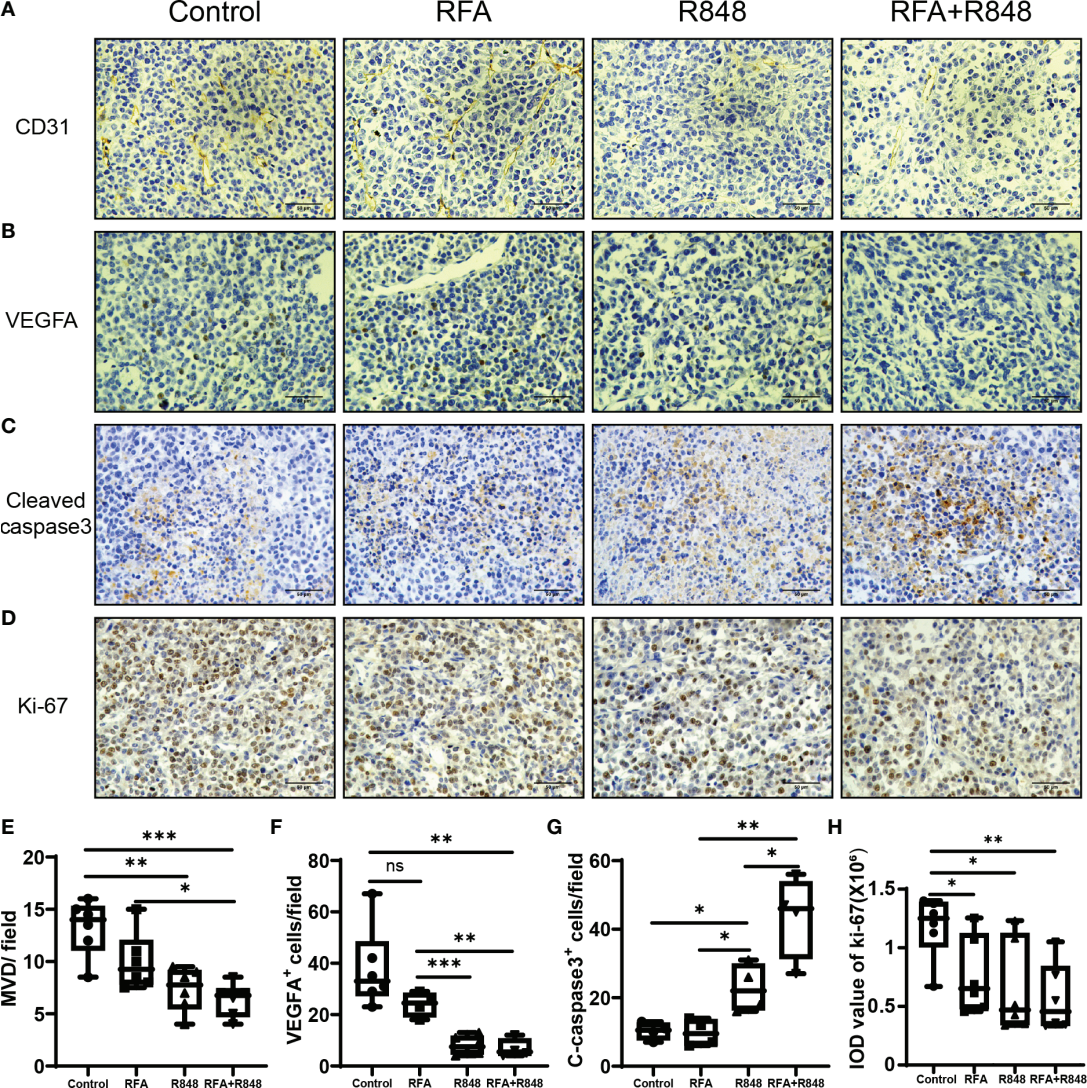
The combination of radiofrequency ablation and resiquimod reduces tumor vascularization and induces apoptosis. Representative images of immunohistochemistry (IHC) staining for CD31 **(A)** and VEGFA **(B)** of tumors collected from mice that were treated with different approaches. **(E, F)** Quantitative analysis of tumor micro-vessel density and the number of VEGFA+ cells per field; *n* = 4–6. **(C, G)** Detection of the expression of apoptosis marker cleaved caspase 3 in tumors by IHC and its corresponding quantitative analysis; *n* = 4. **(D, H)** IHC analysis of the tumor sections stained with Ki67 following different treatments; *n* = 6. Scale bars, 50 μm. The *P*-values were calculated by two-tailed Student’s *t*-test. Error bars represent mean ± SEM. *p < 0.05; **p < 0.01; ***p < 0.001; ns, not significant.

Previous studies have shown that R848 alone can inhibit angiogenesis and promote apoptosis in breast cancer (41). To assess whether R848 plays a similar role in liver cancer, we selected two tumor vascular markers—CD31 and VEGFA—for immunohistochemical staining of tumor tissues. Apparently, the micro-vessel density and VEGFA^+^ cells were decreased in the R848 group and the RFA+R848 group (**Figures 6A** **, B, E, F**), while RFA+R848 did not further reduce the tumor micro-vessels compared with R848 monotherapy. This indicates that the reduction of tumor micro-vessels is mainly caused by R848. In addition, cleaved caspase3 and ki-67 were stained to indicate tumor apoptosis and proliferation. The number of cleaved caspase3^+^ cells in the RFA+R848 group was significantly higher than in the other three groups, and the quantity of cleaved caspase3^+^ cells in the R848 group was also slightly higher than that in the RFA group and the control group (**Figures 6C** **, G**). Meanwhile, both the RFA and R848 treatments have a certain degree of inhibitory effect on tumor proliferation, but no obvious additive effect was observed in the combined treatment (**Figures 6D** **, H**). Overall, these findings are consistent with the changes in tumor size and TIME that we described earlier.

## Discussion

HCC is one of the most common malignant tumors worldwide and remains a global health challenge (9). Local ablation is one of the top three treatment options for HCC, and it is estimated that more than half of HCC patients have received this treatment throughout their lifespan (10). As one of the most commonly used treatment modalities in local ablation, RFA destroys tumor tissues by generating heat through a radiofrequency electrode (16, 42). The heat causes mechanical damage to tumor cells, which, in turn, leads to the release of abundant immunogenic intracellular substrates and damage-associated molecular patterns, such as heat shock proteins, high mobility group protein B1, RNA as well as DNA (16, 43). These elicit a certain degree of anti-tumor immune response as an *in situ* vaccine, but the response is too weak to effectively inhibit tumor progression (10, 42–45). Combined immunotherapy, on the basis of local ablation therapy, is an ideal method to enhance the efficacy of thermal ablation therapy (10, 23, 46). As a novel immunomodulatory agent, R848 has attracted widespread attention in recent years, which plays an important role in promoting the activation of NK cell and CD8^+^ T cell as well as the release of proinflammatory cytokines, such as IFN-α, IL-2, IL-6, and TNF-α (27, 47–49). The purpose of this study was to examine whether RFA in combination with R848 could meaningfully inhibit HCC progression and inflame the TIME.

Some preclinical studies have confirmed that R848 alone can slightly inhibit the progression of pancreatic cancer (27), breast cancer (41), and colorectal cancer (47). Here we demonstrated that RFA combined with R848 further inhibited HCC growth and prolonged the survival time of tumor-bearing mice compared with R848 or RFA monotherapy. This study also revealed the changes in the composition of tumor-infiltrating immune cells by flow cytometry. We found that the total tumor-infiltrating immune cells, the ratio of CD8^+^ T cells and NK cells to the total immune cells, and the percentage of functional NK cells were effectively increased after the combination therapy compared with RFA treatment alone. However, unlike previous studies about TLR7/8 agonist (50–54), our findings suggest that the systemic administration of R848 (1 mg/kg) did not evidently alter the composition of macrophages nor did it effectively promote type I macrophage polarization in murine liver cancer, and the percentage of DC cells in liver cancer and pancreatic cancer (27) was slightly decreased after R848 administration, which is inconsistent with the stronger antitumor immunity induced by R848—so further research is necessary.

Another critical finding of our research is that the combined treatment of RFA and R848 induced a potent abscopal effect. The total tumor-infiltrating immune cells and NK cells in distant tumor tissues were significantly increased. NK cells are abundant in the liver, and studies have shown that the proportion of NK cells in the liver is approximately five times that in the blood and spleen, but it is significantly decreased in the occurrence and development of HCC (34, 55). Moreover, the number of NK cells is positively correlated with the prognosis of HCC patients (56). Previous research had also shown that both RFA (17, 57) and TLR7/8 agonist (58–61) alone can stimulate the activation of NK cells. Our study shows that the proportion of tumor-infiltrating NK cells in both primary and distant tumors of the combined treatment group was higher than that in the RFA or R848 treatment group, suggesting that the combination therapy further promotes the infiltration of NK cells into the HCC and formed a superimposed effect. Furthermore, the NK deletion test clearly proved that the inhibition of liver cancer by the combination therapy is NK cell dependent.

Chemokine networks are critical for driving the intra-tumoral infiltration of NK cells (40). Previous studies have confirmed as well that CXCL10, CX3CL1 (62), and CXCL11 (63) play an important role in the infiltration of NK cells into the melanoma. Correspondingly, we observed that the expression levels of NK cell-related chemokines were the highest in the combined treatment group, especially CCL7, CXCL10, CXCL11, and CX3CL1, which was consistent with the phenomenon that the proportion of NK cells was the highest in the combined treatment group, reflecting that they are responsible for the infiltration of NK cell into the tumor. Moreover, the expression of CXCL11 in the combination therapy group was significantly higher than those in the R848 group, which might be the reason for the additive effect of the combined treatments. However, we have not been able to clarify which chemokine drives the NK cell to infiltrate liver cancer; this needs to be further explored in the future. The expression level of CXCL10 in the RFA group was higher than that in the control group as well. It is a manifestation of the immunogenic cell death of tumor after RFA. On the other hand, recent studies have demonstrated that R848 binding to TLR7/8 stimulates the production of IFN-α, IL-6, IL-12, TNF-α, and other proinflammatory cytokines through MyD88-dependent or MyD88-independent pathway (24, 25, 47), and RFA treatment can also promote the expression of multiple cytokines, such as IL-1β, IL-6, IL-8, and TNF-α (16, 43, 64, 65). Our data shows that the expression levels of IL-2, IL-6, IL-12, TNF-α, and IFN-α/βR in the combination group were significantly higher than those in the RFA or R848 group. This is evidence that RFA and R848 work together to activate anti-tumor immunity. In addition, several studies reported that R848 can inhibit angiogenesis and promote apoptosis in breast cancer (24, 41). Our IHC results certified that the RFA+R848 treatment effectively reduced the vascular density and VEGF expression of liver cancer, but the mechanism of this has not been elucidated; it is an aspect for further research. Besides this, cleaved-caspase3 and ki-67 staining indicated that the combination therapy promoted tumor apoptosis but failed to further inhibit tumor proliferation compared with RFA monotherapy.

In conclusion, this study demonstrated that the combination of RFA with R848 ignited a robust anti-tumor response and significantly constrained HCC progression in a NK cell-dependent manner. Meanwhile, we confirmed that R848 inhibited angiogenesis and promoted apoptosis in murine liver cancer (**Schematic Illustration**). Overall, our research explored the potential of RFA combined with R848 in the treatment of liver cancer and provided a novel insight into the combination of thermal ablation with immunotherapy.

## Data Availability Statement

The datasets presented in this study can be found in online repositories. The names of the repository/repositories and accession number(s) can be found in the article/**Supplementary Material**.

## Ethics Statement

The animal study was reviewed and approved by the Laboratory Animal Welfare Ethics Review Committee of Zhejiang University.

## Author Contributions

ZTi conceived the idea, performed the experiments, and wrote the manuscript. BH provided program guidance. JC and ZTa designed the experiments, directed this study, and revised the manuscript. All authors contributed to the article and approved the submitted version.

## Funding

This work was supported by the Key Research and Development Project of Zhejiang Province (2021C03048), the Key Project of the Natural Science Foundation of Zhejiang Province (LZ20H160002), and the Medical and Health Science and Technology Project of Zhejiang Province (2019KY290 and 2021KY024).

## Conflict of Interest

The authors declare that the research was conducted in the absence of any commercial or financial relationships that could be construed as a potential conflict of interest.

## Publisher’s Note

All claims expressed in this article are solely those of the authors and do not necessarily represent those of their affiliated organizations, or those of the publisher, the editors and the reviewers. Any product that may be evaluated in this article, or claim that may be made by its manufacturer, is not guaranteed or endorsed by the publisher.

## Supplementary Material

Supplementary Figure 1 Radiofrequency ablation, in combination with resiquimod, shrinks the tumor-draining lymph node, but monotherapy does not. Representative pictures to show the tumor-draining lymph node **(A)** and spleen **(B)** size in the mice with different treatments. The quantitative analysis of the TLN and the spleen weight were taken from two independent experiments; n=4-5, **P* < 0.05. Error bars represent mean ± SEM. Statistical analysis was performed by two-tailed Student’s *t*-test.

Supplementary Figure 2 Gating strategy to facilitate the understanding of how different types of cells are defined.

Supplementary Figure 3 The profile of tumor-infiltrating lymphoid immune cells is slightly altered after the combination therapy. **(A**–**C)** The proportion of tumor-infiltrating CD3+, CD4+, CD45R+ cells to total CD45+ immune cells. **(D**–**F)** Cumulated ratio of CD8+ T cells and NK cells expressing IFN-γ or GZMB; *n* = 4. Statistical significance was calculated by two-tailed Student’s *t*-test; **P* < 0.05. Error bars indicate mean ± SEM. GZMB, Granzyme B.

Supplementary Figure 4 Changes in tumor-infiltrating myeloid immune cells after the combination therapy. **(A**–**E)** The frequencies of macrophages (Ly6c-CD11B+F4/80+), monocytes (CD11B+MHCII-Ly6c+Ly6g-), and neutrophils (CD11B+MHCII-Ly6c+Ly6g+) in the total CD45+ immune cells and the ratio of M1 (MHCII+ macrophage) and M2 (MHCII- macrophage) to the total macrophages. **(F**, **G)** The proportion of dendritic cells (DCs, CD11B+MHCII+CD11C+F4/80-) to CD45+ immune cells and the frequencies of CD86+ DCs in total DCs; *n* = 4. Statistical significance was calculated by two-tailed Student’s *t*-test; **P* < 0.05, ***P* < 0.01. All error bars represent mean ± SEM.

Supplementary Figure 5 Distant intra-tumoral T lymphocyte composition is not altered by the combination therapy. Hepa1-6 tumor cells were simultaneously inoculated on the bilateral flanks of immunocompetent C57BL/6 mice; the tumor on the right side is seen as a primary tumor for radiofrequency ablation therapy, and the contralateral tumor was considered as a distant tumor for flow cytometry analysis. resiquimod was injected intraperitoneally once every 2 days for a total of 4 injections. **(A**–**C)** Percentage of CD3+, CD4+, and CD8+ T cells as a percent of CD45+ immune cells in distant tumor; *n* = 4. Statistically significant differences were calculated by two-tailed Student’s *t*-test. All error bars indicate mean ± SEM. ns, no significance.

Supplementary Figure 6 Representative picture of the tumor radiofrequency ablation model.

Supplementary Table 1 List of qPCR primer sequences.

Schematic Illustration Both RFA and R848 can stimulate the production of pro-inflammatory cytokines and chemokines in tumor tissue, thereby stimulating the formation of anti-tumor immunity. The combination of the two treatments further promoted the expression of NK cell-related chemokines and cytotoxic lymphocyte-related cytokines, such as CXCL10, CXCL11, IL2, IFN-γ, TNF-α, *etc.*, which drove lots of NK cells to infiltrate the tumor and enhanced the NK cell function. In addition, R848 inhibited tumor angiogenesis and promoted the apoptosis of liver cancer. As a result, the combination of RFA with R848 improved the anti-tumor immune response induced by RFA and significantly inhibited the progression of liver cancer. RFA, radiofrequency ablation; R848, resiquimod; DAMPs, damage-associated molecular patterns; IICSs, immunogenic intracellular substrates; APC, antigen-presenting cell; NK cell, natural killer cell.
