# Supplementary figures and images for: Combination of Radiofrequency Ablation With Resiquimod to Treat Hepatocellular Carcinoma Via Inflammation of Tumor Immune Microenvironment and Suppression of Angiogenesis
Source: Front Oncol. 2022 Jun 2;12:891724. doi: 10.3389/fonc.2022.891724 (PMC9201999; doi:10.3389/fonc.2022.891724)

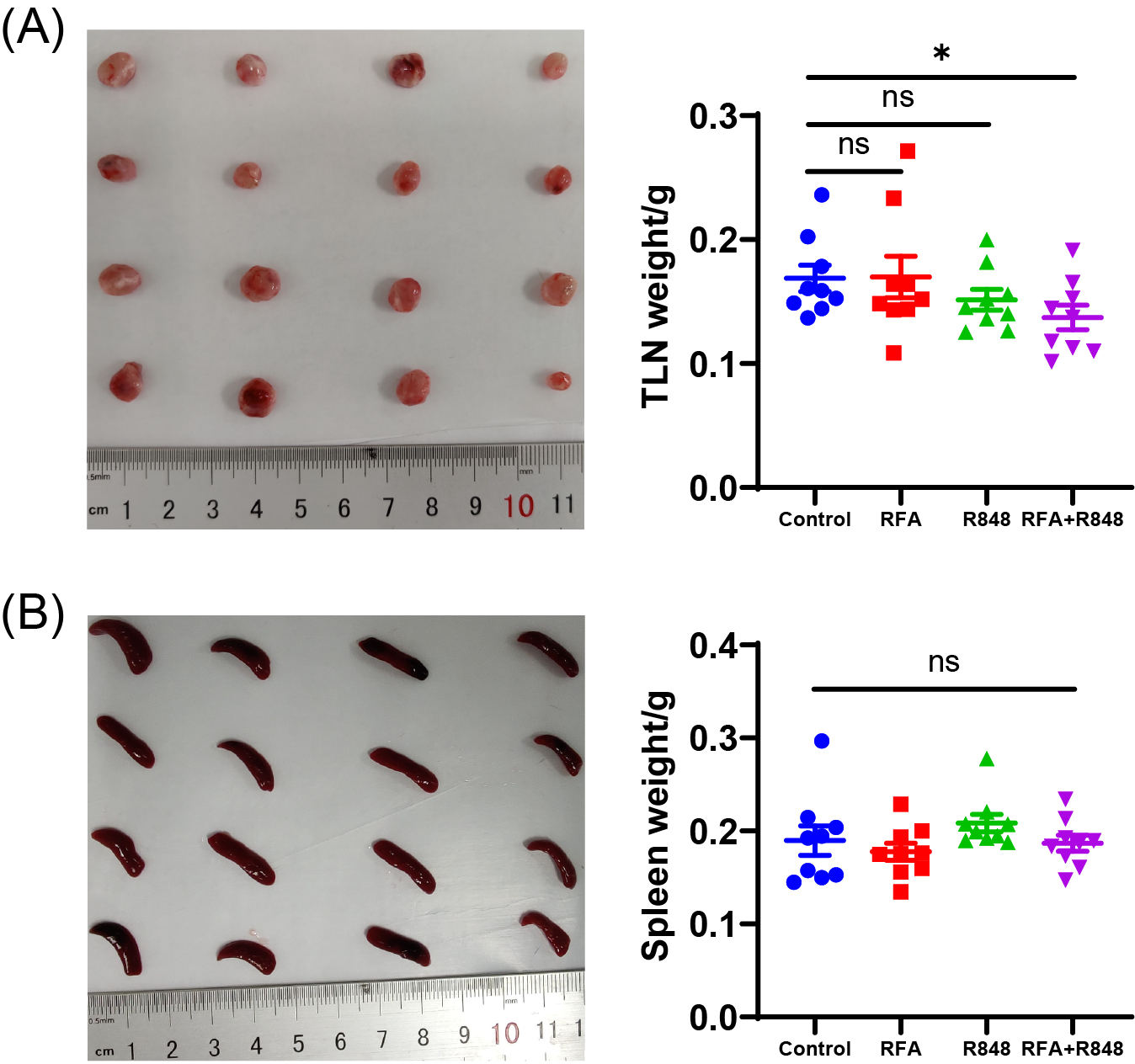

Supplement: Supplementary Figure 1 — Radiofrequency ablation, in combination with resiquimod, shrinks the tumor-draining lymph node, but monotherapy does not. Representative pictures to show the tumor-draining lymph node (A) and spleen (B) size in the mice with different treatments. The quantitative analysis of the TLN and the spleen weight were taken from two independent experiments; n=4-5, *P < 0.05. Error bars represent mean ± SEM. Statistical analysis was performed by two-tailed Student’s t-test. [file Image_1.tif]

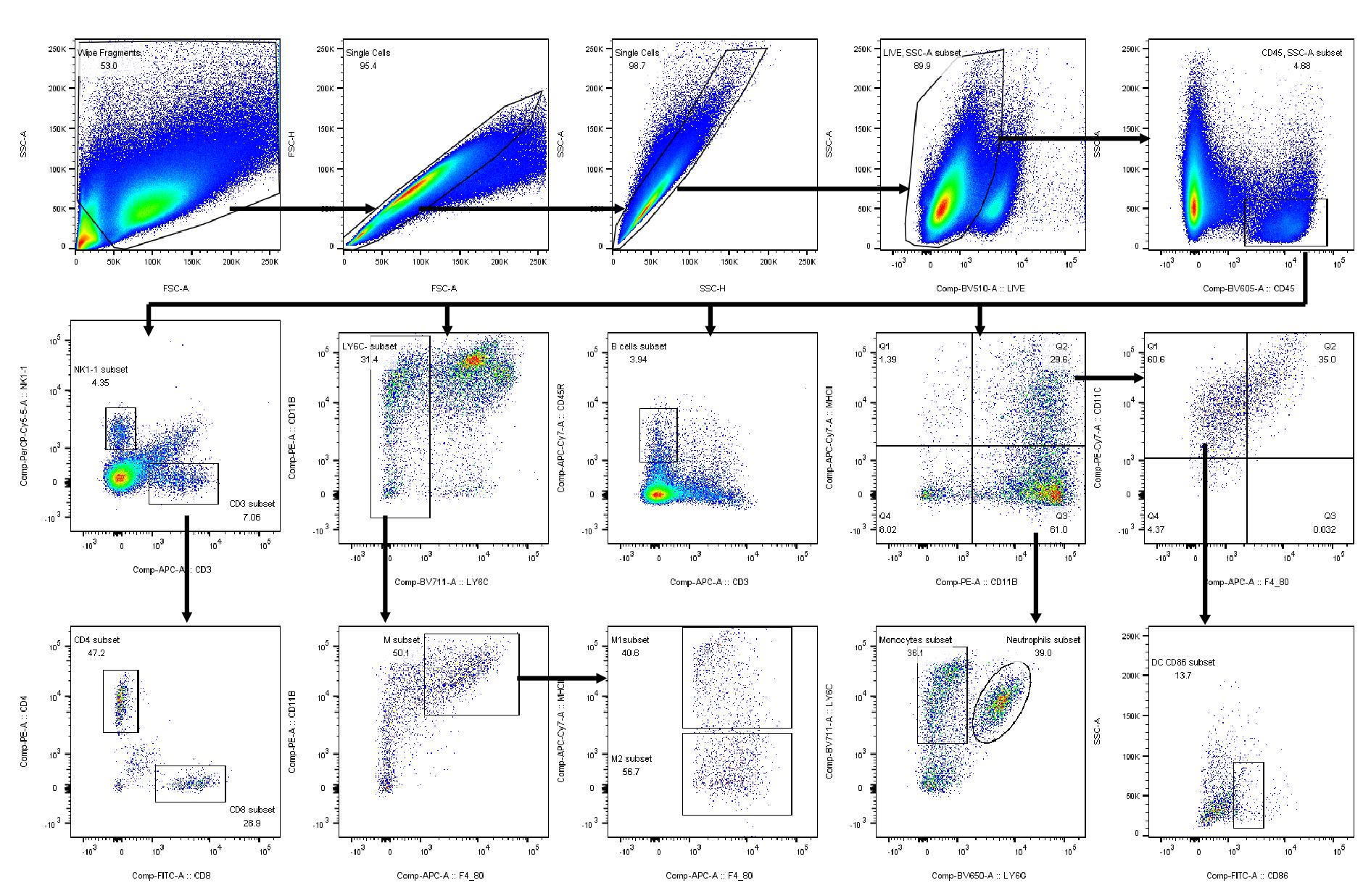

Supplement: Supplementary Figure 2 — Gating strategy to facilitate the understanding of how different types of cells are defined. [file Image_2.tif]

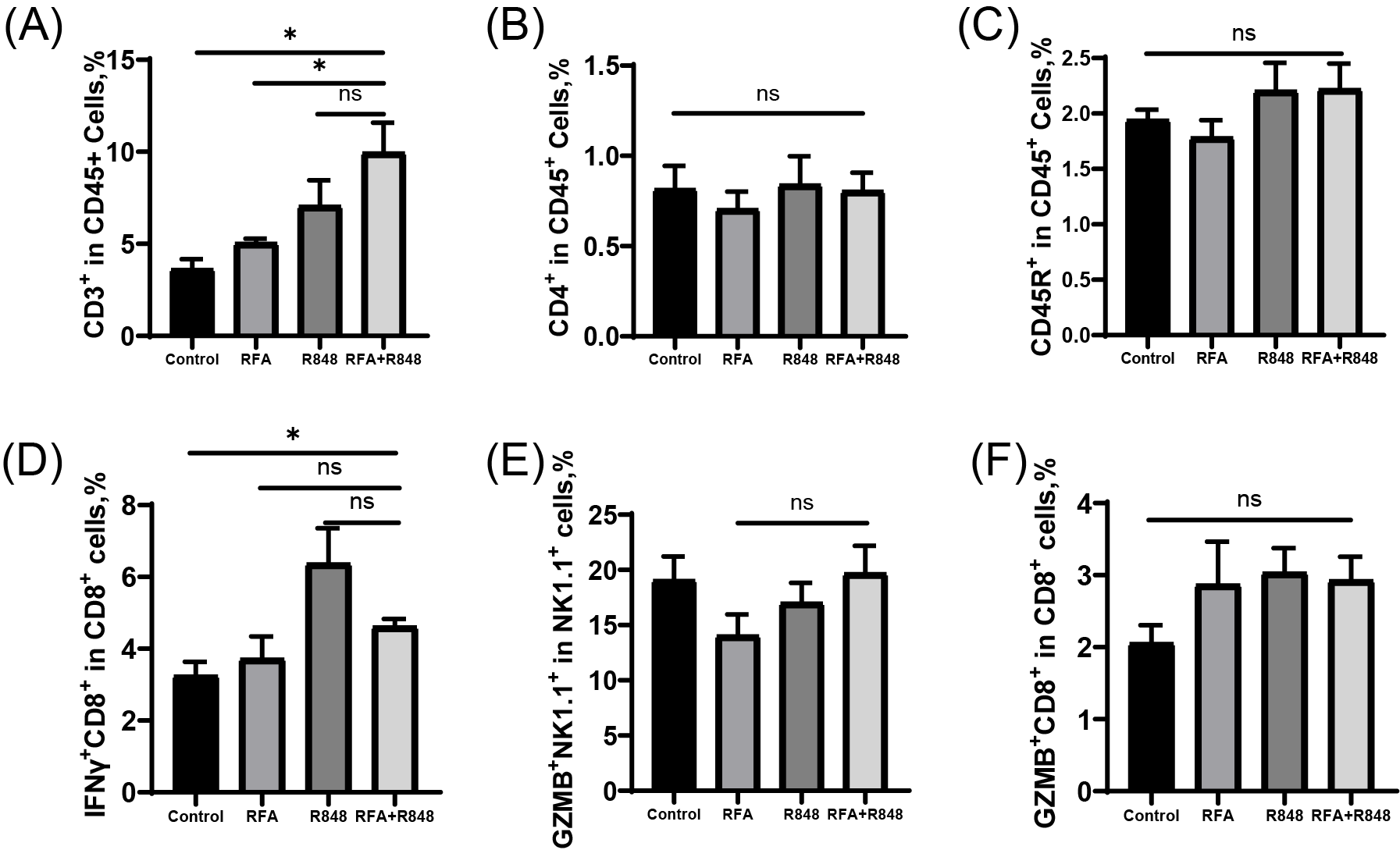

Supplement: Supplementary Figure 3 — The profile of tumor-infiltrating lymphoid immune cells is slightly altered after the combination therapy. (A–C) The proportion of tumor-infiltrating CD3+, CD4+, CD45R+ cells to total CD45+ immune cells. (D–F) Cumulated ratio of CD8+ T cells and NK cells expressing IFN-γ or GZMB; n = 4. Statistical significance was calculated by two-tailed Student’s t-test; *P < 0.05. Error bars indicate mean ± SEM. GZMB, Granzyme B. [file Image_3.tif]

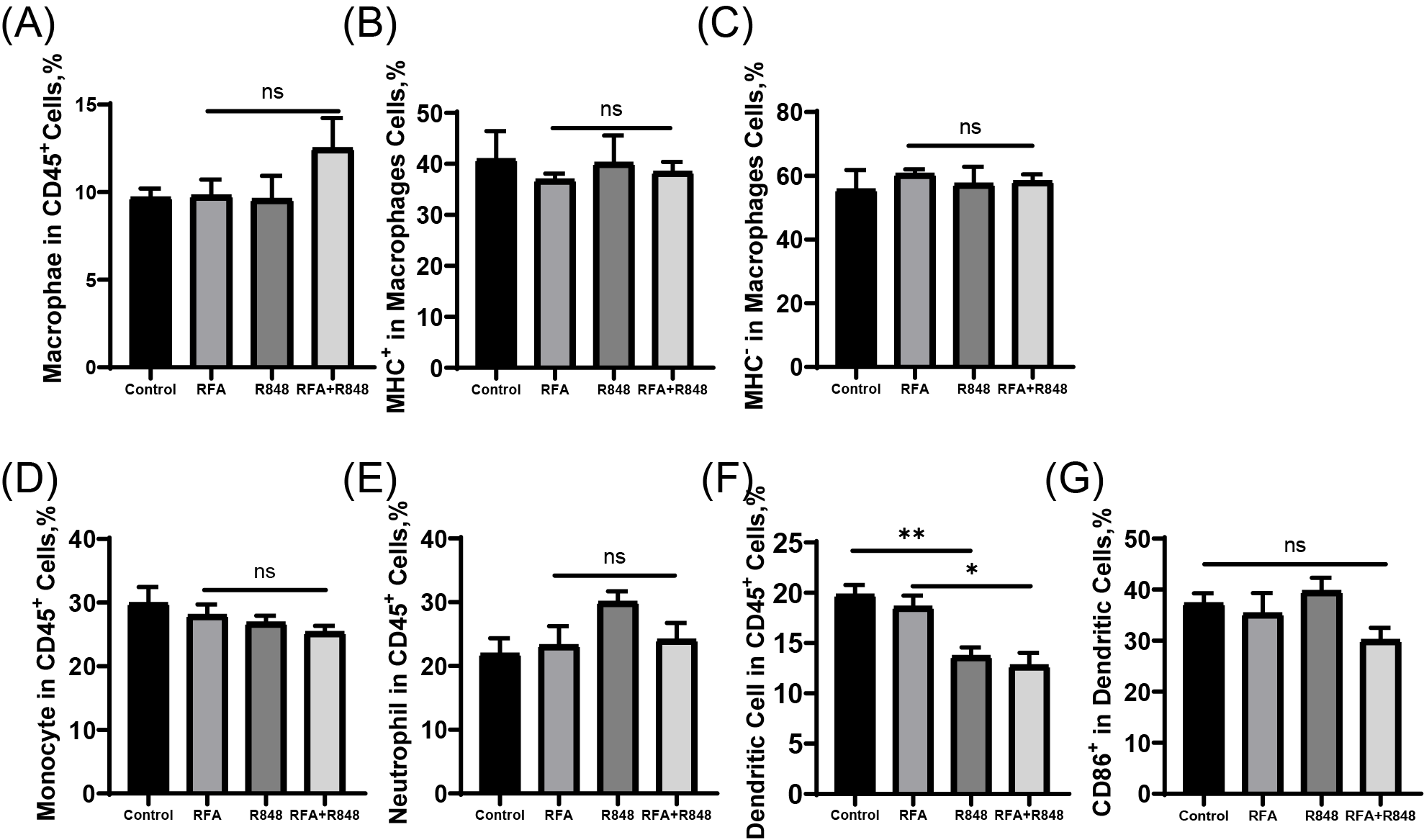

Supplement: Supplementary Figure 4 — Changes in tumor-infiltrating myeloid immune cells after the combination therapy. (A–E) The frequencies of macrophages (Ly6c-CD11B+F4/80+), monocytes (CD11B+MHCII-Ly6c+Ly6g-), and neutrophils (CD11B+MHCII-Ly6c+Ly6g+) in the total CD45+ immune cells and the ratio of M1 (MHCII+ macrophage) and M2 (MHCII- macrophage) to the total macrophages. (F, G) The proportion of dendritic cells (DCs, CD11B+MHCII+CD11C+F4/80-) to CD45+ immune cells and the frequencies of CD86+ DCs in total DCs; n = 4. Statistical significance was calculated by two-tailed Student’s t-test; *P < 0.05, **P < 0.01. All error bars represent mean ± SEM. [file Image_4.tif]

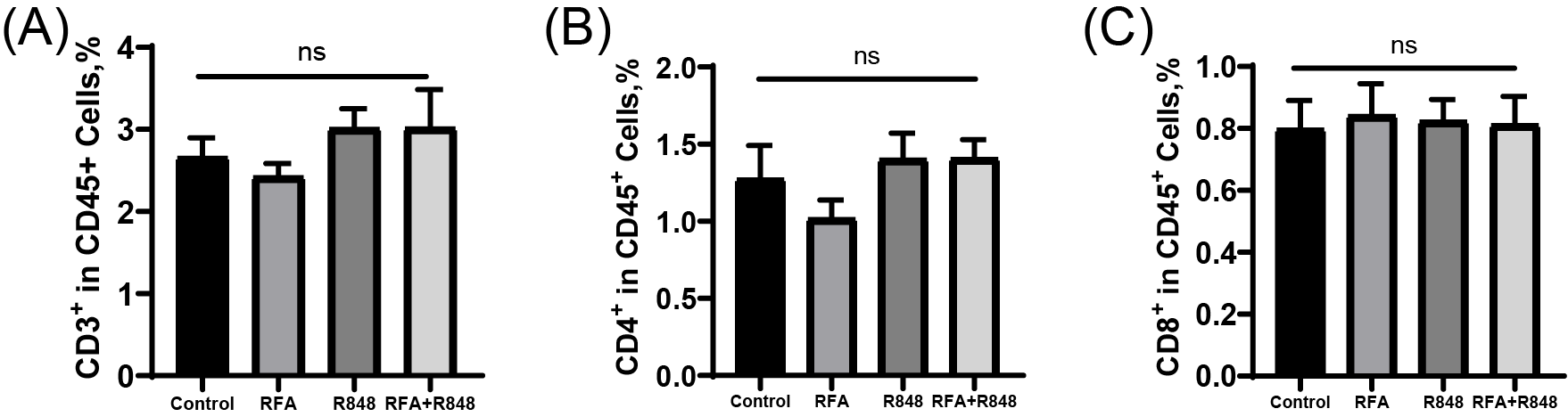

Supplement: Supplementary Figure 5 — Distant intra-tumoral T lymphocyte composition is not altered by the combination therapy. Hepa1-6 tumor cells were simultaneously inoculated on the bilateral flanks of immunocompetent C57BL/6 mice; the tumor on the right side is seen as a primary tumor for radiofrequency ablation therapy, and the contralateral tumor was considered as a distant tumor for flow cytometry analysis. resiquimod was injected intraperitoneally once every 2 days for a total of 4 injections. (A–C) Percentage of CD3+, CD4+, and CD8+ T cells as a percent of CD45+ immune cells in distant tumor; n = 4. Statistically significant differences were calculated by two-tailed Student’s t-test. All error bars indicate mean ± SEM. ns, no significance. [file Image_5.tif]

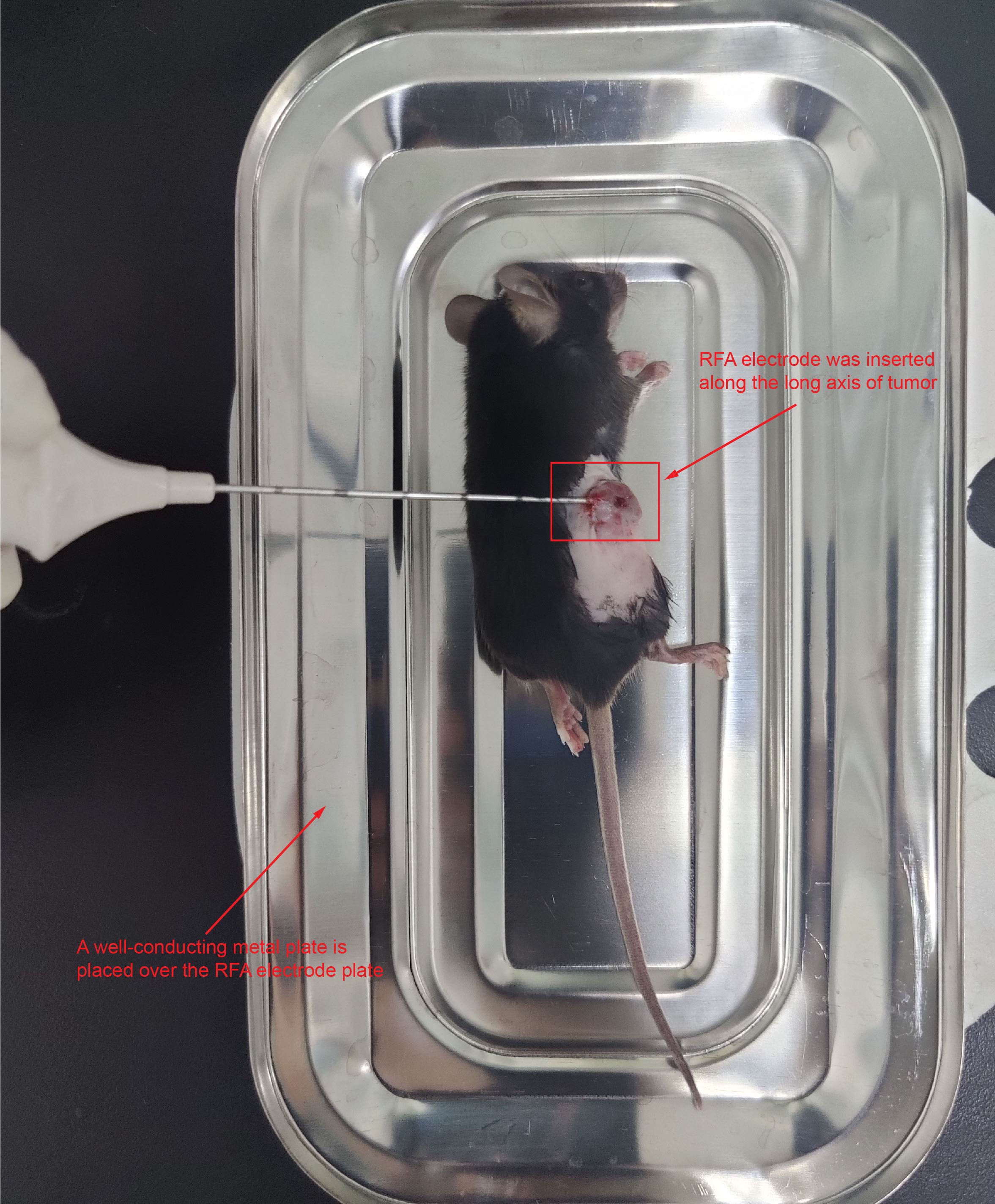

Supplement: Supplementary Figure 6 — Representative picture of the tumor radiofrequency ablation model. [file Image_6.jpeg]

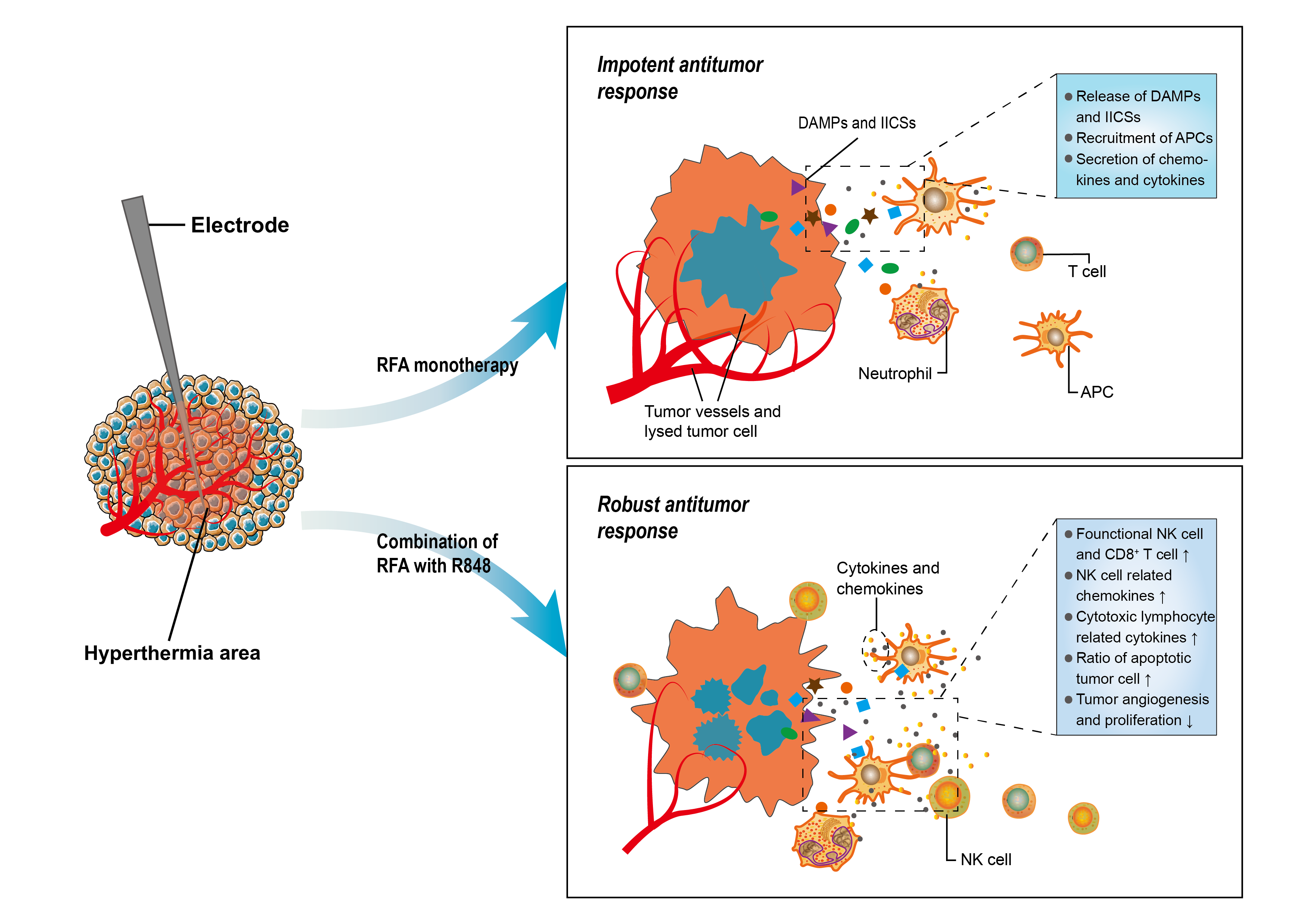

Supplement: Supplementary file 8 [file DataSheet_1.zip › Figure 7.tif]
