# Supplementary material for: Combination of Radiofrequency Ablation With Resiquimod to Treat Hepatocellular Carcinoma Via Inflammation of Tumor Immune Microenvironment and Suppression of Angiogenesis
Source: Front Oncol. 2022 Jun 2;12:891724. doi: 10.3389/fonc.2022.891724 (PMC9201999; doi:10.3389/fonc.2022.891724)
Supplement: Supplementary Table 1 — List of qPCR primer sequences. [file Table_1.docx]

**Supplementary Table 1**

| List of qPCR primer sequences | | |
| --- | --- | --- |
| Target | Forward primer (5' to 3') | Reverse primer (5' to 3') |
| IL-2 | TGAGCAGGATGGAGAATTACAGG | GTCCAAGTTCATCTTCTAGGCAC |
| IL-6 | AGTTGCCTTCTTGGGACTGA | TCCACGATTTCCCAGAGAAC |
| IL-12 | CTGTGCCTTGGTAGCATCTATG | GCAGAGTCTCGCCATTATGATTC |
| TNF-α | CAGGCGGTGCCTATGTCTC | CGATCACCCCGAAGTTCAGTAG |
| IFN-α/β | AGCCACGGAGAGTCAATGG | GCTCTGACACGAAACTGTGTTTT |
| IFN-γ | ATGAACGCTACACACTGCATC | CCATCCTTTTGCCAGTTCCTC |
| CCL2 | TTAAAAACCTGGATCGGAACCAA | GCATTAGCTTCAGATTTACGGGT |
| CCL5 | GCTGCTTTGCCTACCTCTCC | TCGAGTGACAAACACGACTGC |
| CCL7 | GCTGCTTTCAGCATCCAAGTG | CCAGGGACACCGACTACTG |
| CCL8 | TCTACGCAGTGCTTCTTTGCC | AAGGGGGATCTTCAGCTTTAGTA |
| CCL19 | GGGGTGCTAATGATGCGGAA | CCTTAGTGTGGTGAACACAACA |
| CXCL9 | TCCTTTTGGGCATCATCTTCC | TTTGTAGTGGATCGTGCCTCG |
| CXCL10 | CCAAGTGCTGCCGTCATTTTC | GGCTCGCAGGGATGATTTCAA |
| CXCL11 | GGCTTCCTTATGTTCAAACAGGG | GCCGTTACTCGGGTAAATTACA |
| CX3CL1 | ACGAAATGCGAAATCATGTGC | CTGTGTCGTCTCCAGGACAA |
| Actin | AACAGTCCGCCTAGAAGCAC | CGTTGACATCCGTAAAGACC |
